# 10-Ethynyl-2,3,6,6a,9,10-hexa­hydro-1*H*-6,9-methano­pyrrolo[2,1-*i*][2,1]benzo­thia­zol-10-ol 5,5-dioxide

**DOI:** 10.1107/S1600536809038410

**Published:** 2009-10-03

**Authors:** B. O. Patrick, H. Liang, S. Canesi, M. A. Ciufolini

**Affiliations:** a Department of Chemistry, University of British Columbia, 2036 Main Mall, Vancouver, BC, Canada V6T 1Z1

## Abstract

In the title compound, C_13_H_15_NO_3_S, the sole classical hydrogen-bond donor is involved in an intra­molecular O—H⋯N hydrogen bond. In the crystal structure, pairs of mol­ecules related by inversion centres are linked by pairs of weak inter­molecular C—H⋯O inter­actions; these centrosymmetric pairs are, in turn, linked further by weak inter­molecular C—H⋯O inter­actions, forming two-dimensional sheets oriented parallel to (101).

## Related literature

For background to our ongoing research on the synthesis of himandrine and related alkaloids, see: Ciufolini *et al.* (2007 ▶); Liang & Ciufolini (2008 ▶).
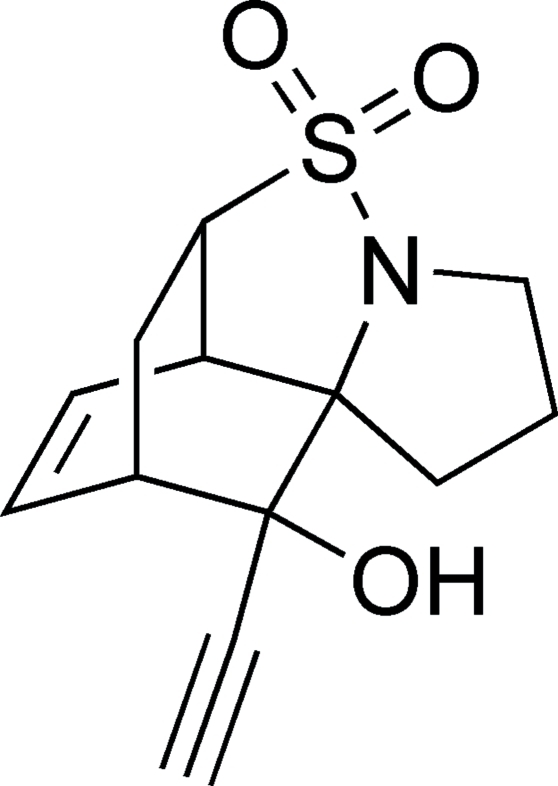


## Experimental

### 

#### Crystal data

C_13_H_15_NO_3_S
*M* *_r_* = 265.32
Monoclinic, 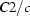

*a* = 24.113 (3) Å
*b* = 6.6202 (7) Å
*c* = 15.111 (2) Å
β = 92.625 (5)°
*V* = 2409.6 (5) Å^3^
*Z* = 8
Mo *K*α radiation
μ = 0.27 mm^−1^
*T* = 173 K
0.35 × 0.27 × 0.18 mm

#### Data collection

Bruker X8 APEXII diffractometer
Absorption correction: multi-scan (*SADABS*; Bruker, 2008 ▶) *T*_min_ = 0.877, *T*_max_ = 0.963
13946 measured reflections
2889 independent reflections
2523 reflections with *I* > 2σ(*I*)
*R*_int_ = 0.030

#### Refinement

*R*[*F* ^2^ > 2σ(*F* ^2^)] = 0.034
*wR*(*F* ^2^) = 0.095
*S* = 1.03
2889 reflections
167 parameters
H atoms treated by a mixture of independent and constrained refinement
Δρ_max_ = 0.33 e Å^−3^
Δρ_min_ = −0.42 e Å^−3^

### 

Data collection: *APEX2* (Bruker, 2008 ▶); cell refinement: *SAINT* (Bruker, 2008 ▶); data reduction: *SAINT*; program(s) used to solve structure: *SIR97* (Altomare *et al.*, 1999 ▶); program(s) used to refine structure: *SHELXL97* (Sheldrick, 2008 ▶); molecular graphics: *ORTEP-3 for Windows* (Farrugia, 1997 ▶); software used to prepare material for publication: *WinGX* (Farrugia, 1999 ▶).

## Supplementary Material

Crystal structure: contains datablocks I, global. DOI: 10.1107/S1600536809038410/lh2908sup1.cif

Structure factors: contains datablocks I. DOI: 10.1107/S1600536809038410/lh2908Isup2.hkl

Additional supplementary materials:  crystallographic information; 3D view; checkCIF report

## Figures and Tables

**Table 1:** Hydrogen-bond geometry (Å, °)

*D*—H⋯*A*	*D*—H	H⋯*A*	*D*⋯*A*	*D*—H⋯*A*
O9—H9*O*⋯N13	0.83 (2)	1.99 (2)	2.606 (1)	131 (2)
C8—H8⋯O16^i^	1.00	2.53	3.183 (2)	123
C18—H18⋯O9^ii^	0.95	2.40	3.341 (2)	169

Symmetry codes: (i) 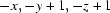; (ii) 
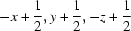
.

**Fig. 1.**
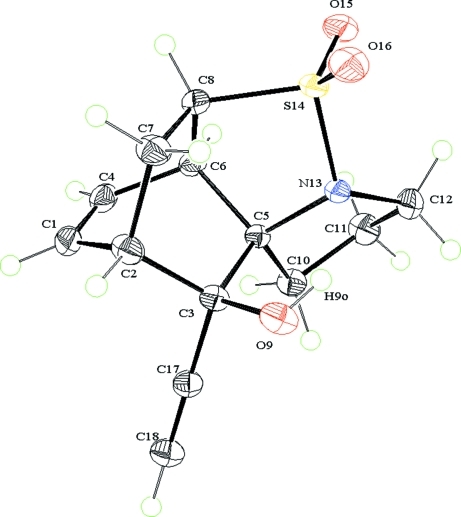
The molecular structure of the title compound, with atom labels and 50% probability displacement ellipsoids for non-H atoms.

**Fig. 2.**
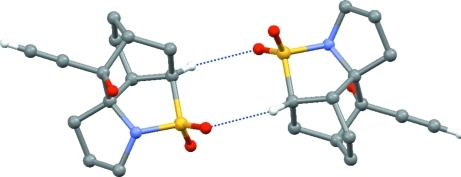
A centrosymmetric pair of molecules with weak intermolecular C—H···O interactions shown as dashed lines.

**Fig. 3.**
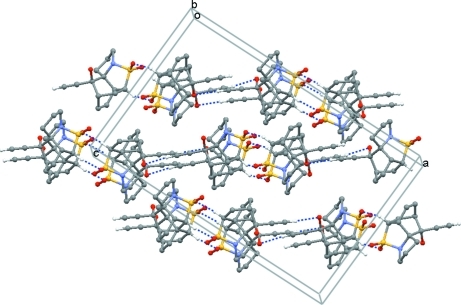
Part of the crystal structure of the title compound, showing C-H···O hydrogen-bonded (dashed lines) sheets parallel to the (101) plane.

## supplementary crystallographic information

### Comment

The oxidative amidation of phenols offers interesting opportunities in the synthesis of nitrogenous substances. We employed spirocyclization of phenolic sulfonamides to prepare a tricyclic intermediate in the ongoing research on the synthesis of himandrine and related alkaloids (Liang *et al.*, 2008; Ciufolini *et al.*,  2007). The molecular stucture of the title compound is shown in Fig.1. In the crystal structure, pairs of molecules for related by inversion centres are linked by weak intermolecular C—H···O interactions (Table 1, Fig. 2). These centrosymmetric pairs, are in turn, linked further by weak intermolecular C—H···O interactions to form 2-D sheets oriented parallel to the (101) plane, as shown in Fig.3.

### Experimental

Potassium carbonate (137 mg, 0.99 mmol) was added to a solution of 10- [(trimethylsilyl)ethynyl]-2,3,6,6a,9,10-hexahydro-1*H*-6,9-methanopyrrolo [2,1-*i*][2,1]benzisothiazol-10-ol 5,5-dioxide (110 mg, 0.33 mmol) in MeOH (1 ml). Upon the completion of the reaction, the mixture was concentrated and dried over high vacuum. Chromatography of the residue (EtOAc / hexanes = 1 / 2) gave 78 mg (0.29 mmol, 89%) product as a colourless solid. X-ray quality single crystals were obtained by slow evaporation of a dichloromethane/hexanes (1:2*v*/*v*) solution of the title compound over two weeks.

### Refinement

H atoms boned to C atoms were placed in calculated positions with C-H = 0.93-1.00Å and included in the refinement with U_iso_(H) = 1.2U_eq_(C). The hydroxyl H atom was refined indpendently with an isotropic displacement parameter.

### Crystal data

**Table:**

C_13_H_15_NO_3_S	*F*(000) = 1120
*M**_r_* = 265.32	*D*_x_ = 1.463 Mg m^−^^3^
Monoclinic, *C*2/*c*	Mo *K*α radiation, λ = 0.71073 Å
Hall symbol: -C 2yc	Cell parameters from 6461 reflections
*a* = 24.113 (3) Å	θ = 2.7–28.1°
*b* = 6.6202 (7) Å	µ = 0.27 mm^−^^1^
*c* = 15.111 (2) Å	*T* = 173 K
β = 92.625 (5)°	Prism, colourless
*V* = 2409.6 (5) Å^3^	0.35 × 0.27 × 0.18 mm
*Z* = 8	

### Data collection

**Table:**

Bruker X8 APEXII diffractometer	2889 independent reflections
Radiation source: fine-focus sealed tube	2523 reflections with *I* > 2σ(*I*)
graphite	*R*_int_ = 0.030
φ and ω scans	θ_max_ = 28.0°, θ_min_ = 1.7°
Absorption correction: multi-scan (*SADABS*; Bruker, 2008)	*h* = −31→30
*T*_min_ = 0.877, *T*_max_ = 0.963	*k* = −7→8
13946 measured reflections	*l* = −19→19

### Refinement

**Table:**

Refinement on *F*^2^	Primary atom site location: structure-invariant direct methods
Least-squares matrix: full	Secondary atom site location: difference Fourier map
*R*[*F*^2^ > 2σ(*F*^2^)] = 0.034	Hydrogen site location: inferred from neighbouring sites
*wR*(*F*^2^) = 0.095	H atoms treated by a mixture of independent and constrained refinement
*S* = 1.03	*w* = 1/[σ^2^(*F*_o_^2^) + (0.0543*P*)^2^ + 1.8974*P*] where *P* = (*F*_o_^2^ + 2*F*_c_^2^)/3
2889 reflections	(Δ/σ)_max_ = 0.001
167 parameters	Δρ_max_ = 0.33 e Å^−^^3^
0 restraints	Δρ_min_ = −0.42 e Å^−^^3^

### Special details

**Table:**

Geometry. All e.s.d.'s (except the e.s.d. in the dihedral angle between two l.s. planes) are estimated using the full covariance matrix. The cell e.s.d.'s are taken into account individually in the estimation of e.s.d.'s in distances, angles and torsion angles; correlations between e.s.d.'s in cell parameters are only used when they are defined by crystal symmetry. An approximate (isotropic) treatment of cell e.s.d.'s is used for estimating e.s.d.'s involving l.s. planes.
Refinement. Refinement of *F*^2^ against ALL reflections. The weighted *R*-factor *wR* and goodness of fit *S* are based on *F*^2^, conventional *R*-factors *R* are based on *F*, with *F* set to zero for negative *F*^2^. The threshold expression of *F*^2^ > σ(*F*^2^) is used only for calculating *R*-factors(gt) *etc*. and is not relevant to the choice of reflections for refinement. *R*-factors based on *F*^2^ are statistically about twice as large as those based on *F*, and *R*- factors based on ALL data will be even larger.

### Fractional atomic coordinates and isotropic or equivalent isotropic displacement parameters (Å^2^)

**Table:**

	*x*	*y*	*z*	*U*_iso_*/*U*_eq_	
C1	0.08386 (6)	0.8014 (2)	0.24477 (10)	0.0260 (3)	
H1	0.0804	0.8017	0.1819	0.031*	
C2	0.10447 (6)	0.6218 (2)	0.29734 (9)	0.0227 (3)	
H2	0.1137	0.5077	0.2573	0.027*	
C3	0.15678 (6)	0.69210 (19)	0.35283 (9)	0.0167 (3)	
C4	0.07087 (6)	0.9590 (2)	0.29339 (10)	0.0241 (3)	
H4	0.0580	1.0826	0.2681	0.029*	
C5	0.13832 (5)	0.86156 (19)	0.41981 (8)	0.0143 (2)	
C6	0.07809 (5)	0.9277 (2)	0.39197 (9)	0.0179 (3)	
H6	0.0669	1.0507	0.4252	0.021*	
C7	0.05869 (6)	0.5588 (2)	0.36090 (10)	0.0246 (3)	
H7A	0.0251	0.5144	0.3261	0.030*	
H7B	0.0720	0.4446	0.3985	0.030*	
C8	0.04457 (6)	0.7400 (2)	0.41948 (9)	0.0192 (3)	
H8	0.0038	0.7682	0.4163	0.023*	
C10	0.17913 (6)	1.0368 (2)	0.43416 (9)	0.0197 (3)	
H10A	0.2177	0.9926	0.4252	0.024*	
H10B	0.1698	1.1497	0.3931	0.024*	
C11	0.17199 (6)	1.0997 (2)	0.53035 (10)	0.0258 (3)	
H11A	0.2046	1.1761	0.5543	0.031*	
H11B	0.1382	1.1827	0.5361	0.031*	
C12	0.16678 (6)	0.8971 (2)	0.57668 (9)	0.0238 (3)	
H12A	0.2038	0.8389	0.5919	0.029*	
H12B	0.1462	0.9112	0.6315	0.029*	
C17	0.19816 (6)	0.7687 (2)	0.29199 (9)	0.0187 (3)	
C18	0.23235 (6)	0.8204 (2)	0.24254 (10)	0.0237 (3)	
H18	0.2597	0.8618	0.2030	0.028*	
N13	0.13541 (5)	0.76842 (17)	0.51039 (7)	0.0166 (2)	
O9	0.18201 (4)	0.52655 (15)	0.39984 (7)	0.0242 (2)	
O15	0.04568 (4)	0.85899 (19)	0.58703 (7)	0.0300 (3)	
O16	0.06809 (5)	0.50367 (17)	0.56167 (8)	0.0316 (3)	
S14	0.069910 (13)	0.70937 (5)	0.53140 (2)	0.01917 (11)	
H9O	0.1739 (10)	0.542 (3)	0.4521 (16)	0.049 (6)*	

### Atomic displacement parameters (Å^2^)

**Table:**

	*U*^11^	*U*^22^	*U*^33^	*U*^12^	*U*^13^	*U*^23^
C1	0.0192 (7)	0.0402 (9)	0.0183 (7)	−0.0073 (6)	−0.0010 (5)	0.0025 (6)
C2	0.0242 (7)	0.0233 (7)	0.0210 (7)	−0.0070 (6)	0.0053 (5)	−0.0054 (5)
C3	0.0182 (6)	0.0136 (6)	0.0187 (6)	0.0007 (5)	0.0044 (5)	0.0015 (5)
C4	0.0178 (6)	0.0313 (8)	0.0228 (7)	0.0008 (6)	−0.0017 (5)	0.0094 (6)
C5	0.0148 (6)	0.0128 (6)	0.0156 (6)	0.0005 (5)	0.0023 (4)	0.0017 (5)
C6	0.0152 (6)	0.0180 (6)	0.0205 (6)	0.0021 (5)	0.0012 (5)	0.0031 (5)
C7	0.0235 (7)	0.0255 (7)	0.0252 (7)	−0.0096 (6)	0.0052 (6)	−0.0040 (6)
C8	0.0149 (6)	0.0239 (7)	0.0188 (6)	−0.0023 (5)	0.0015 (5)	0.0018 (5)
C10	0.0200 (6)	0.0163 (6)	0.0228 (7)	−0.0038 (5)	0.0026 (5)	−0.0006 (5)
C11	0.0277 (7)	0.0236 (7)	0.0261 (7)	−0.0062 (6)	0.0012 (6)	−0.0059 (6)
C12	0.0223 (7)	0.0293 (8)	0.0194 (7)	−0.0024 (6)	−0.0020 (5)	−0.0013 (6)
C17	0.0187 (6)	0.0164 (6)	0.0209 (6)	0.0019 (5)	0.0022 (5)	−0.0003 (5)
C18	0.0231 (7)	0.0232 (7)	0.0252 (7)	−0.0003 (5)	0.0067 (6)	0.0016 (6)
N13	0.0147 (5)	0.0194 (6)	0.0160 (5)	−0.0001 (4)	0.0027 (4)	0.0027 (4)
O9	0.0301 (5)	0.0163 (5)	0.0271 (6)	0.0078 (4)	0.0097 (4)	0.0062 (4)
O15	0.0232 (5)	0.0426 (7)	0.0248 (5)	0.0037 (5)	0.0081 (4)	−0.0073 (5)
O16	0.0286 (6)	0.0307 (6)	0.0357 (6)	−0.0060 (5)	0.0048 (5)	0.0151 (5)
S14	0.01614 (17)	0.0232 (2)	0.01853 (18)	−0.00074 (12)	0.00492 (12)	0.00346 (12)

### Geometric parameters (Å, °)

**Table:**

C1—C4	1.322 (2)	C7—H7B	0.9900
C1—C2	1.501 (2)	C8—S14	1.7832 (14)
C1—H1	0.9500	C8—H8	1.0000
C2—C7	1.5532 (19)	C10—C11	1.529 (2)
C2—C3	1.5536 (19)	C10—H10A	0.9900
C2—H2	1.0000	C10—H10B	0.9900
C3—O9	1.4269 (16)	C11—C12	1.521 (2)
C3—C17	1.4770 (18)	C11—H11A	0.9900
C3—C5	1.5882 (17)	C11—H11B	0.9900
C4—C6	1.5062 (19)	C12—N13	1.4938 (18)
C4—H4	0.9500	C12—H12A	0.9900
C5—N13	1.5057 (16)	C12—H12B	0.9900
C5—C10	1.5304 (18)	C17—C18	1.188 (2)
C5—C6	1.5563 (17)	C18—H18	0.9500
C6—C8	1.5497 (18)	N13—S14	1.6716 (11)
C6—H6	1.0000	O9—H9O	0.83 (2)
C7—C8	1.538 (2)	O15—S14	1.4402 (11)
C7—H7A	0.9900	O16—S14	1.4378 (11)
			
C4—C1—C2	114.33 (13)	C7—C8—C6	109.82 (11)
C4—C1—H1	122.8	C7—C8—S14	112.45 (10)
C2—C1—H1	122.8	C6—C8—S14	100.68 (9)
C1—C2—C7	108.20 (12)	C7—C8—H8	111.2
C1—C2—C3	106.82 (11)	C6—C8—H8	111.2
C7—C2—C3	109.22 (11)	S14—C8—H8	111.2
C1—C2—H2	110.8	C11—C10—C5	103.97 (11)
C7—C2—H2	110.8	C11—C10—H10A	111.0
C3—C2—H2	110.8	C5—C10—H10A	111.0
O9—C3—C17	106.79 (11)	C11—C10—H10B	111.0
O9—C3—C2	110.84 (11)	C5—C10—H10B	111.0
C17—C3—C2	108.77 (11)	H10A—C10—H10B	109.0
O9—C3—C5	110.54 (10)	C12—C11—C10	102.28 (11)
C17—C3—C5	111.79 (10)	C12—C11—H11A	111.3
C2—C3—C5	108.13 (10)	C10—C11—H11A	111.3
C1—C4—C6	114.92 (13)	C12—C11—H11B	111.3
C1—C4—H4	122.5	C10—C11—H11B	111.3
C6—C4—H4	122.5	H11A—C11—H11B	109.2
N13—C5—C10	103.78 (10)	N13—C12—C11	104.12 (11)
N13—C5—C6	106.21 (10)	N13—C12—H12A	110.9
C10—C5—C6	114.26 (11)	C11—C12—H12A	110.9
N13—C5—C3	108.43 (10)	N13—C12—H12B	110.9
C10—C5—C3	115.39 (10)	C11—C12—H12B	110.9
C6—C5—C3	108.17 (10)	H12A—C12—H12B	109.0
C4—C6—C8	109.72 (12)	C18—C17—C3	176.65 (15)
C4—C6—C5	111.73 (11)	C17—C18—H18	180.0
C8—C6—C5	101.16 (10)	C12—N13—C5	109.44 (10)
C4—C6—H6	111.3	C12—N13—S14	117.37 (9)
C8—C6—H6	111.3	C5—N13—S14	110.62 (8)
C5—C6—H6	111.3	C3—O9—H9O	105.3 (16)
C8—C7—C2	109.18 (11)	O16—S14—O15	116.53 (7)
C8—C7—H7A	109.8	O16—S14—N13	108.95 (6)
C2—C7—H7A	109.8	O15—S14—N13	111.24 (6)
C8—C7—H7B	109.8	O16—S14—C8	113.28 (7)
C2—C7—H7B	109.8	O15—S14—C8	110.17 (7)
H7A—C7—H7B	108.3	N13—S14—C8	94.53 (6)
			
C4—C1—C2—C7	58.42 (16)	C5—C6—C8—C7	−67.18 (13)
C4—C1—C2—C3	−59.07 (15)	C4—C6—C8—S14	169.71 (9)
C1—C2—C3—O9	−175.33 (11)	C5—C6—C8—S14	51.57 (10)
C7—C2—C3—O9	67.86 (14)	N13—C5—C10—C11	−29.30 (13)
C1—C2—C3—C17	−58.21 (14)	C6—C5—C10—C11	85.90 (13)
C7—C2—C3—C17	−175.02 (12)	C3—C5—C10—C11	−147.76 (11)
C1—C2—C3—C5	63.37 (13)	C5—C10—C11—C12	40.51 (14)
C7—C2—C3—C5	−53.44 (14)	C10—C11—C12—N13	−35.77 (14)
C2—C1—C4—C6	−1.13 (18)	O9—C3—C17—C18	35 (3)
O9—C3—C5—N13	−18.73 (14)	C2—C3—C17—C18	−85 (3)
C17—C3—C5—N13	−137.54 (11)	C5—C3—C17—C18	156 (3)
C2—C3—C5—N13	102.76 (11)	C11—C12—N13—C5	18.13 (14)
O9—C3—C5—C10	97.12 (13)	C11—C12—N13—S14	−108.98 (11)
C17—C3—C5—C10	−21.69 (16)	C10—C5—N13—C12	6.98 (13)
C2—C3—C5—C10	−141.39 (11)	C6—C5—N13—C12	−113.80 (12)
O9—C3—C5—C6	−133.51 (11)	C3—C5—N13—C12	130.14 (11)
C17—C3—C5—C6	107.68 (12)	C10—C5—N13—S14	137.81 (9)
C2—C3—C5—C6	−12.02 (13)	C6—C5—N13—S14	17.02 (12)
C1—C4—C6—C8	−55.26 (16)	C3—C5—N13—S14	−99.03 (10)
C1—C4—C6—C5	56.10 (17)	C12—N13—S14—O16	−103.72 (11)
N13—C5—C6—C4	−161.09 (11)	C5—N13—S14—O16	129.74 (9)
C10—C5—C6—C4	85.14 (14)	C12—N13—S14—O15	26.08 (12)
C3—C5—C6—C4	−44.87 (14)	C5—N13—S14—O15	−100.46 (9)
N13—C5—C6—C8	−44.42 (12)	C12—N13—S14—C8	139.77 (10)
C10—C5—C6—C8	−158.19 (11)	C5—N13—S14—C8	13.23 (10)
C3—C5—C6—C8	71.81 (12)	C7—C8—S14—O16	−34.96 (11)
C1—C2—C7—C8	−56.73 (15)	C6—C8—S14—O16	−151.79 (9)
C3—C2—C7—C8	59.20 (15)	C7—C8—S14—O15	−167.50 (9)
C2—C7—C8—C6	3.27 (16)	C6—C8—S14—O15	75.67 (10)
C2—C7—C8—S14	−107.96 (12)	C7—C8—S14—N13	77.91 (10)
C4—C6—C8—C7	50.96 (15)	C6—C8—S14—N13	−38.92 (9)

### Hydrogen-bond geometry (Å, °)

**Table:**

*D*—H···*A*	*D*—H	H···*A*	*D*···*A*	*D*—H···*A*
O9—H9O···N13	0.83 (2)	1.99 (2)	2.606 (1)	131 (2)
C8—H8···O16^i^	1.00	2.53	3.183 (2)	123
C18—H18···O9^ii^	0.95	2.40	3.341 (2)	169

Symmetry codes: (i) −*x*, −*y*+1, −*z*+1; (ii) −*x*+1/2, *y*+1/2, −*z*+1/2.
